# Analytical Parameter Estimation of the SIR Epidemic Model. Applications to the COVID-19 Pandemic

**DOI:** 10.3390/e23010059

**Published:** 2020-12-31

**Authors:** Dimiter Prodanov

**Affiliations:** 1 Environment, Health and Safety, Leuven, IMEC, Kapeldreef 75, 3001 Leuven, Belgium; dimiter.prodanov@imec.be; 2 MMSDP, IICT, Bulgarian Academy of Sciences, Acad. G. Bonchev St., Block 25A, 1113 Sofia, Bulgaria

**Keywords:** SIR model, special functions, lambert W function, wright omega function

## Abstract

The SIR (Susceptible-Infected-Removed) model is a simple mathematical model of epidemic outbreaks, yet for decades it evaded the efforts of the mathematical community to derive an explicit solution. The present paper reports novel analytical results and numerical algorithms suitable for parametric estimation of the SIR model. Notably, a series solution of the incidence variable of the model is derived. It is proven that the explicit solution of the model requires the introduction of a new transcendental special function, describing the incidence, which is a solution of a non-elementary integral equation. The paper introduces iterative algorithms approximating the incidence variable, which allows for estimation of the model parameters from the numbers of observed cases. The approach is applied to the case study of the ongoing coronavirus disease 2019 (COVID-19) pandemic in five European countries: Belgium, Bulgaria, Germany, Italy and the Netherlands. Incidence and case fatality data obtained from the European Centre for Disease Prevention and Control (ECDC) are analysed and the model parameters are estimated and compared for the period Jan-Dec 2020.

## 1. Introduction

The dramatic outbreak of the coronavirus disease 2019 (COVID-19) pandemics and its ongoing progression boosted the scientific community’s interest in epidemic modelling and forecasting. The coronavirus 2019 (COVID-19) disease was reported to appear for the first time in Wuhan, China, and later it spread to Europe, which is the subject of the presented case studies, and eventually worldwide. While there are individual clinical reports for COVID-19 re-infections, the present stage of the pandemic still allows for the application of a relatively simple epidemic model, which is the subject of the present report. The motivation behind the presented research was the intention to accurately model the short-term dynamics of the outbreaks of COVID-19 pandemics, which by the time of the first submission of the present article, has infected more than 27 million individuals worldwide. The efforts to contain the spread of the pandemic induce sustained social and economic damage. Therefore, the ability to accurately forecast short to medium-term epidemic outbreak’s dynamics is of substantial public interest.

The present paper focuses on the utility of the SIR (Susceptible-Infected-Removed) epidemiological model in epidemic outbreak modelling. The SIR model was introduced in 1927 by Kermack and McKendrick in 1927 to study the plague and cholera epidemics in London and Bombay [1]. The original paper also introduced some asymptotes of the model variables. To date the SIR model remains as a cornerstone of mathematical epidemiology. It is a deterministic model formulated in terms of ordinary differential equations (ODEs). The model has been extensively used to study the spread of various infectious diseases (see the monograph of Martcheva [2]). Outside epidemiology, the SIR model is also extensively used in modelling of online social networks, viral marketing, diffusion of ideas, spread of computer viruses, financial network contagion, etc. (see recent survey by Rodrigues and the references therein [3]).

The SIR model can be modified in several ways, for example by including demographics, deceased population (i.e., SIRD) or hidden populations (i.e., “exposed” individuals–SEIR) [2]. As the conditions of an accelerating epidemic outbreak often make contact tracing problematic, the SEIR model needs to make additional assumptions. Therefore, outcome variables, such as mortality, can be foretasted more easily using a simpler model, e.g., using the SIR model. Recently, several authors demonstrated that the first wave of COVID-19 followed SIR dynamic, which by itself, is an interesting finding [4,5,6,7,8,9].

The present paper has two main objectives—(i) to report some new analytical results about SIR model and (ii) to introduce an algorithm for the estimation of the parameters of the SIR model from empirical time series data. A challenge with the forecasting, based on empirical estimation of the parameters, is the inherent uncertainty built in such estimates. This combined with the multiplicative interactions in the SIR model variables can lead to huge discrepancies between the observed and forecasted values. While the SIR model has been modified to include also other sub-populations the empirical estimation of the model parameters poses even greater challenge. In contrast to previous approaches, the paper does not approach the SIR model as an initial value problem but as a problem in the theory of special functions. This change of perspective allows for relative ease of handling of noisy data, e.g., time series having fluctuations caused by delays and accumulation of case reporting. The numerical approach was applied to the COVID-19 incidence and case fatality data from different European countries, having different population densities and dynamics of the epidemic outbreaks. The methods of the present paper go beyond the presented COVID-19 application case and can be used in a variety of fields as indicated above.

## 2. The SIR Model

The SIR model is formulated in terms of three populations of individuals. The *S* population consists of all individuals susceptible to the infection of concern. The *I* population comprises the infected individuals. These persons have the disease and can transmit it to the susceptible individuals. The *R* population represents the immune individuals, who cannot become infected and cannot transmit the disease to others. The model comprises a system of three ODEs:

(1) $$\begin{array}{cc} \dot{S}\left(t\right) & =-\frac{\beta }{N}S\left(t\right)I\left(t\right) \end{array}$$

(2) $$\begin{array}{cc} \dot{I}\left(t\right) & =\frac{\beta }{N}S\left(t\right)I\left(t\right)-\gamma I\left(t\right) \end{array}$$

(3) $$\begin{array}{cc} \dot{R}\left(t\right) & =\gamma I(t) \end{array}$$

The model assumes a constant overall population N=S+I+R. A disease carrier infects on average β individuals per day, for an average time of 1/γ days. The β parameter is called *disease transmission rate*, while γ—*recovery rate*. The average number of infections arising from an infected individual is then modelled by the number $R_{0}=\frac{\beta }{\gamma }$, the *basic reproduction number*. Typical initial conditions are $S\left(0\right)=S_{0},\;I\left(0\right)=I_{0},\;R\left(0\right)=0$ [1].

The model can be re-parametrized using normalized variables as

(4) $$\begin{array}{cc} \dot{s} & =-si \end{array}$$

(5) $$\begin{array}{cc} \dot{i} & =si-gi,\;g=\frac{\gamma }{\beta }=\frac{1}{R_{0}} \end{array}$$

(6) $$\begin{array}{cc} \dot{r} & =gi, \end{array}$$

subject to normalization s+i+r=1 and time rescaling τ=βt. This is a model in dimensionless time τ. It this way, *g* also becomes a dimensionless parameter. Since i(τ) is integrable on [0,∞) then i(∞)=0.

## 3. The Implicit Analytical Solution

The analytical solution will be formulated first in an implicit form. Since there is a first integral by construction the system can be reduced to two equations:

(7) $$\begin{array}{cc} \frac{di}{ds} & =-1+\frac{g}{s} \end{array}$$

(8) $$\begin{array}{cc} \frac{di}{dr} & =\frac{s}{g}-1 \end{array}$$

**Remark** **1.** *From this formulation*

$$R_{e}=N\frac{s_{0}}{g}=\frac{S_{0}\beta }{\gamma }\geq 1$$

*must hold for the infection to propagate. $R_{e}$ is called the effective reproductive number, while the basic reproduction number is $R_{0}=R_{e}N$ [10].*

In order to solve the model we will consider the two equations separately. Direct integration of Equation (7) gives

i=−s+glogs+c

where the constant *c* can be determined from the initial conditions. In the present treatment, the constant *c* will be left indeterminate to be assigned by the different re-parametrization procedures. The *s* variable can be expressed explicitly in terms of the Lambert W function [11]:

(9) $$s=-gW_{\pm }\left(-\frac{e^{\frac{i-c}{g}}}{g}\right)$$

where the signs denote the two different real-valued branches of the function. Note, that both branches are of interest since the argument of the Lambert W function is negative. Therefore, Equation (5) can be reduced to the first-order autonomous system

(10) $$\dot{i}=-ig\left(W_{\pm }\left(-\frac{e^{\frac{i-c}{g}}}{g}\right)+1\right)$$

valid for two disjoined domains on the real line. The equation can be solved for the time τ by quadrature as

(11) $$-\int \frac{di}{i\left(W_{\pm }\left(-\frac{e^{\frac{i-c}{g}}}{g}\right)+1\right)}=g\tau$$

**Remark** **2.** *There is another equivalent form of the system using the Wright *Ω* function [12] since*

$$W\left(-\frac{e^{\frac{i-c}{g}}}{g}\right)=\Omega \left(\frac{i-c}{g}-\log\left(-g\right)\right)$$

*so that*

$$\dot{i}=-ig\left(\Omega \left(\frac{i-c-g\log g}{g}\pm j\pi \right)+1\right)$$

*where j represents the imaginary unit.*

The *s* variable can be determined by substitution in Equation (4), resulting in the autonomous system

(12) $$\dot{s}=-s\left(-s+g\log s+c\right)$$

which can be solved implicitly as

(13) $$\int \frac{ds}{s\left(s-g\log s-c\right)}=\tau$$

**Remark** **3.** *This implicit solution for the s-variable has been derived originally in [13]. Note that in the present case it was not necessary to derive and solve a Bernoulli differential equation [13,14]. Therefore, this is a new method of proof. The authors did not establish the non-elementary character of the integral. The results for the i-variable are original.*

Finally, the *r* variable can also be conveniently expressed in terms of *i*. For this purpose we solve the differential equation

$$\frac{dr}{di}=\frac{g}{s-g}=\frac{-1}{1+W_{\pm }\left(-\frac{e^{\frac{i-c}{g}}}{g}\right)}$$

Therefore,

$$r=c_{1}-g\log\left(-gW_{\pm }\left(-\frac{e^{\frac{i-c}{g}}}{g}\right)\right)$$

by Proposition A4. On the other hand,

$$\begin{array}{c} g\log\left(-gW\left(-\frac{e^{\frac{i-c}{g}}}{g}\right)\right)=g\;\left(\log\left(\frac{e^{\frac{i-c}{g}}}{g}\right)-W\left(-\frac{e^{\frac{i-c}{g}}}{g}\right)\right)=\\ -gW\left(-\frac{e^{\frac{i-c}{g}}}{g}\right)+i-g\log g-c=s+i-g\log g-c \end{array}$$

So that

$$r=gW\left(-\frac{e^{\frac{i-c}{g}}}{g}\right)-i+c_{1}$$

For the purposes of curve fitting we assume that i(−∞)=r(−∞)=0. Therefore,

$$c_{1}=-gW_{-}\left(-\frac{e^{-\frac{c}{g}}}{g}\right)$$

### 3.1. Peak Value Parametrization

The peak-value parametrization is necessary for the treatment of the problem as a special function. The initial value parametrization is sensitive to the noise in the initial value conditions, and is therefore unsuitable for curve-fitting. The upper terminal of integration can be determined by the requirement for the real-valuedness of *i*. This value of *i* is denoted as $i_{m}$; that is

$$W_{\pm }\left(-\frac{e^{\frac{i_{m}-c}{g}}}{g}\right)=-1$$

Therefore,

(14) $$i_{m}=c-g\log g-g$$

The peak-value parametrization is supported by the fact that i(t) attains a global maximum $i=i_{m}=c-g\log g-g$ Proposition (A1).

If we consider formally the phase space $\left(z\times y=-gz\left(W_{\pm }\left(-e^{\frac{z-i_{m}}{g}-1}\right)+1\right)\right)$ the following argument allows for the correct branch identification. For i→−∞ it follows that $W_{-}\left(-e^{\frac{z-i_{m}}{g}-1}\right)$ →−∞ so y<0; while $W_{+}\left(-e^{\frac{z-i_{m}}{g}-1}\right)\to 0^{+}$ so y>0. Therefore, if we move the origin as $t\left(0\right)=i_{m}$ then conveniently

(15) $$\begin{array}{cc} -\int_{i_{m}}^{i}\frac{dz}{z\left(W_{+}\left(-e^{\frac{z-i_{m}}{g}-1}\right)+1\right)} & =g\tau ,\;\tau >0 \end{array}$$

(16) $$\begin{array}{cc} -\int_{i_{m}}^{i}\frac{dz}{z\left(W_{-}\left(-e^{\frac{z-i_{m}}{g}-1}\right)+1\right)} & =g\tau ,\;\tau \leq 0 \end{array}$$

Furthermore, the recovered population under this parametrization is

(17) $$r=gW_{\pm }\left(-e^{\frac{i-i_{m}}{g}-1}\right)-gW_{-}\left(-e^{\frac{-i_{m}}{g}-1}\right)-i$$

under the same choice of origin.

### 3.2. Initial Value Parametrization

As customarily accepted, the SIR model can be solved as an initial value problem. The indeterminate constant *c* can be eliminated using the initial condition

$$i_{0}=-s_{0}+g\log s_{0}+c$$

Therefore,

(18) $$i=i_{0}+s_{0}-s+g\log s/s_{0}=1-s+g\log\frac{s}{1-i_{0}}$$

For this case, the following autonomous differential equation can be formulated:

(19) $$\dot{i}=-gi\left(W_{\pm }\left(-\frac{1-i_{0}}{g}e^{\frac{i-1}{g}}\right)+1\right)$$

This can be solved implicitly by separation of variables as

(20) $$\begin{array}{c} -\int_{i_{0}}^{i}\frac{dz}{z\left(W_{-}\left(-\frac{1-i_{0}}{g}e^{\frac{z-1}{g}}\right)+1\right)}=g\tau ,\;\tau \leq t_{m} \end{array}$$

(21) $$\begin{array}{c} -\int_{i_{0}}^{i}\frac{dz}{z\left(W_{+}\left(-\frac{1-i_{0}}{g}e^{\frac{z-1}{g}}\right)+1\right)}=g\tau ,\;\tau >t_{m} \end{array}$$

It is noteworthy that the time to the peak of infections $t_{m}$ can be calculated as

(22) $$t_{m}=\overset{\log g/s_{0}}{\underset{0}{\int }}\frac{du}{s_{0}e^{u}-gu-\left(s_{0}+i_{0}\right)}$$

The result follows by considering the autonomous system Equation (7) and fixing the upper terminal of integration s=g. However, by Proposition A3 this definite integral can be evaluated only numerically.

## 4. Is the Incidence Function “New”?

The incidence *i*-function of the SIR model appears to be an interesting object of study on its own. One may pose the question about the representation of this function by other, possibly, elementary functions. The answer to this question is in the negative as will be demonstrated below. In precise terms, this function is non-Liouvillian. However, this does not mean that the function can not be well approximated. Fortunately, this is the case as the *i*-function can be approximated for a sufficiently wide domain of parameter values by Newtonian iteration.

**Definition** **1.** *An elementary function is defined as a function built from a finite number of combinations and compositions of algebraic, exponential and logarithm functions under algebraic operations (+,−,., /)*

Allowing for the underlying field to be complex numbers—C, trigonometric functions become elementary as well.

**Definition** **2** (Liouvillian function). *We say that f(x) is a Liouvillian function if it lies in some Liouvillian extension of $\left(C\left(x\right){,}^{\prime }\right)$ for some constant field C.*

As a first point we establish the non-elementary character of the integral in Equation (11). The necessary introduction to the theory of differential fields is given in the Appendix C. From the work of Liouville it is known that if a function $F\left(x\right)=f\left(x\right)e^{q(x)}$, where *f*, *q* are elementary functions, has an elementary anti-derivative of the form [15]

$$\int F\left(x\right)dx=\int fe^{q}dx=he^{q}$$

for some elementary function h(x) [16]. Therefore, differentiating we obtain

$$fe^{q}=h^{\prime }e^{q}+hq^{\prime }e^{q}$$

so that if $e^{q}\neq 0$

$$h^{\prime }+hq^{\prime }=f$$

holds. The claim can be strengthened to demand that *h* be algebraic for algebraic *f* and *q*.

**Theorem** **1.** *The integrals*

$$I_{\pm }\left(\xi \right)=\int \frac{d\xi }{\xi \left(W_{\pm }\left(-\frac{e^{\frac{\xi -c}{g}}}{g}\right)+1\right)}$$

*are not Liouvillian.*

**Proof.**  We use $i_{m}$ parametrization. Let $c=i_{m}+g-g\log g$. The proof proceeds by change of variables – first $\xi =g\log y-yg+g+i_{m}$; followed by $z=\log\left(\left(g\log y+i_{m}+g\right)/g\right)$.

$$\begin{array}{c} I=\int \frac{d\xi }{\xi \left(W_{\pm }\left(-e^{\frac{\xi -i_{m}}{g}-1}\right)+1\right)}=\int \frac{y-1}{y\left(g\log\left(y\right)-gy+i_{m}+g\right)\left(W\left(-ye^{-y}\right)+1\right)}dy\\ =-\int \frac{dy}{y(g\log\left(y\right)-gy+i_{m}+g)}=\frac{1}{g}\int \left.\frac{e^{z+\frac{i_{m}}{g}+1}}{e^{e^{z}}-e^{z+\frac{i_{m}}{g}+1}}dz\right. \end{array}$$

since $W_{\pm }\left(-ye^{-y}\right)=-y$. The last integral has the form

$$\int \frac{Ae^{z}}{e^{e^{z}}-Ae^{z}}dz$$

which allows for the application of the Liouville theorem in the form of Corollary A1. We can identify

$$\int fe^{z}dx=he^{z},\;f\left(z\right)=\frac{A}{e^{e^{z}}-Ae^{z}},\;A=e^{\frac{i_{m}}{g}+1}$$

so that

$$\frac{A}{e^{e^{z}}-Ae^{z}}=h^{\prime }\left(z\right)+h\left(z\right)$$

for some unknown algebraic h(z). Since the left-hand side of the equation is transcendental in *z* so is the right-hand side. Therefore, the integrand has no elementary antiderivative. ☐

**Theorem** **2.** *The incidence function i(t), defined by the differential Equation (10), is not Liouvillian.*

**Proof.**  For the present case, let us assume that $i\left(0\right)=i_{m}$ so that *i* attains the maximum by Proposition A1. Therefore,

$$i^{\prime }=-gi\left(W\left(-e^{\frac{i-i_{m}}{g}-1}\right)+1\right)$$

Without loss of generality let g=1, which amounts to scaling of the solution by the factor of 1/g.

Suppose further that

$$i-i_{m}-1=\log u-u$$

for some algebraic function *u* (log-extension case). Then

$$-\frac{i^{\prime }}{i}=W\left(-e^{\log u-u}\right)+1=-u+1$$

On the other hand,

$$-\frac{i^{\prime }}{i}=\log{\left(\log u-u+i_{m}+1\right)}^{\prime }=-\frac{1-u}{u\left(\log u-u+i_{m}+1\right)}u^{\prime }$$

so that

$$-\frac{1-u}{u\left(\log u-u+i_{m}+1\right)}=\frac{1-u}{u^{\prime }}$$

However, if *u* is algebraic so is $u^{\prime }$ by Theorem A1. Therefore, we have a contradiction, since the left-hand-side is transcendental. Hence, *i* is not part of a logarithmic elementary extension.

Suppose that *u* is exponential, i.e., $u=e^{f}$ for some algebraic function *f*. In this case,

$$\frac{\left(e^{f}-1\right)\;f^{\prime }}{-e^{f}+f+i_{m}+1}=1-e^{f}\to -f^{\prime }=-e^{f}+f+i_{m}+1$$

However,

$$f^{\prime }+f+i_{m}+1=e^{f}$$

Therefore, the right-hand-side is exponential and can not be algebraic as it is demanded by Corollary A1. This is a contradiction, hence *f* can not be algebraic, hence *u* is not part of an exponential elementary extension.

Finally, suppose that i(t) is algebraic. Since the Wright function Ω(z) is transcendental [12] it follows that $W\left(-e^{i-i_{m}-1}\right)=\Omega \left(i-i_{m}-1+i\pi \right)$ can not be algebraic in *i*. Therefore, $i^{\prime }/i$ and hence *i* must be transcendental. Hence, the case of an algebraic i(t) can not hold either.

In summary all three cases are rejected, therefore, *i* is not Liouvillian. ☐

## 5. Explicit Series Solutions

We will derive two types of series for the *i*-function in view of the different re-parametrizations of the SIR model. The series can be derived starting from Equation (10) and applying the Taylor theorem.

### 5.1. Series for the $i_{m}$ Parametrization

The natural parametrization is fixing the peak at the origin. The Taylor development can be computed as follows:

(23) $$i\left(t\right)=i_{m}-\frac{{i_{m}}^{2}g}{2}t^{2}+\frac{{i_{m}}^{3}g\;}{6}t^{3}+\frac{\left(4{i_{m}}^{3}g^{2}-{i_{m}}^{4}g\right)}{24}t^{4}-\frac{\left(15{i_{m}}^{4}g^{2}-{i_{m}}^{5}g\right)}{120}t^{5}+\ldots$$

and for the logarithm

(24) $$\log i(t)=\log i_{m}-\frac{i_{m}g}{2}t^{2}+\frac{{i_{m}}^{2}g}{6}t^{3}+\frac{\left({i_{m}}^{2}g^{2}-{i_{m}}^{3}g\right)}{24}t^{4}-\frac{\left(5{i_{m}}^{3}g^{2}-{i_{m}}^{4}g\right)}{120}t^{5}+\ldots$$

### 5.2. Series for the $i_{0}$-Parametrization

The Taylor series starting from an initial value $i_{0}$ is

(25) $$\begin{array}{c} i\left(t\right)=i_{0}-i_{0}\left(i_{0}+g-1\right)t+\frac{g\;i_{0}\left(4{i_{0}}^{2}+5g\;i_{0}-7i_{0}-3g+3\right)}{2\left(i_{0}-1\right)}t^{2}\\ -\frac{g\;i_{0}\left(5{i_{0}}^{3}+21g\;{i_{0}}^{2}-9{i_{0}}^{2}+18g^{2}\;i_{0}-26g\;i_{0}+4i_{0}-10g^{2}+7g\right)}{6\left(i_{0}-1\right)}t^{3}+\ldots \end{array}$$

The Taylor series for the logarithm starting from an initial value $i_{0}$ is

(26) $$\begin{array}{c} \log i(t)=\log\left(i_{0}\right)-\left(i_{0}+g-1\right)t+\frac{g\;i_{0}\left(i_{0}+2g-1\right)}{2\left(i_{0}-1\right)}t^{2}\\ -\frac{g\;i_{0}\left(i_{0}+2g-1\right)\left(2i_{0}+g-1\right)}{6\left(i_{0}-1\right)}t^{3}+\ldots \end{array}$$

The series follow directly from successive differentiation of the differential Equation (19).

## 6. Numerical Approximation

The i-function can be efficiently approximated by the Newton’s method. The Newton iteration scheme is given as follows for the c-parametrization:

$$i_{n+1}=i_{n}-i_{n}\;\left(W_{\pm }\left(-\frac{e^{\frac{i_{n}-c}{g}}}{g}\right)+1\right)\;\left(\int_{g\log g-g+c}^{i_{n}}\left.\frac{d\xi }{\xi \;\left(W_{\pm }\left(-\frac{e^{\frac{\xi -c}{g}}}{g}\right)+1\right)}\right.+gt\right)$$

This is a conceptually simple representation. However, it has the disadvantage of using the Lambert function inside the quadrature routine. Another equivalent representation is

$$i_{n+1}=i_{n}+gi_{n}\;\left(W_{\pm }\left(-\frac{e^{\frac{i_{n}-c}{g}}}{g}\right)+1\right)\;\left(t-\int_{g}^{-gW_{\pm }\left(-\frac{e^{\frac{i_{n}-c}{g}}}{g}\right)}\left.\frac{dy}{y\;\left(g\log y-y+c\right)}\right.\right)$$

(see Proposition A5). This form has the advantage of requiring only 1 Lambert function evaluation per iteration and using only elementary function in the quadrature routine.

A point of attention here is the choice of the initial value for the iteration scheme. Despite the present author’s best efforts, a rigorous analytical asymptotic valid on the entire real line and for all parameter values could not be found. Numerical experiments gave acceptable results using the formula

$$f\left(x\right)=b\;e^{1-xc-e^{-xc}}$$

$g>0,\;c=\sqrt{2bg}$ or $c=2\sqrt{bg/e}$, and additionally $c\leftarrow c/\sqrt{e},\;x>0$ for the initial value of $i_{0}=f\left(t\right)$.

The analytical formula involving first exponentiation and then computation of the Lambert W function has disadvantages for large arguments due to float under or overflows [12]. Therefore, another special function can be used in principle, notably the Wright Ω function. On the other hand, optimized routines for its calculation are not readily available in MATLAB.

### Plots

**Figure 1:**
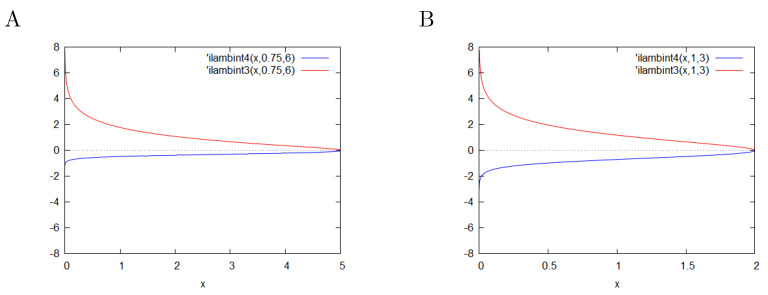
Plots of the integrals $I_{\pm }\left(x\right)$. (**A**)—c-parametrization with parameters g = 0.75, c = 6.0; (**B**)—c-parametrization with parameters g = 1.0, c = 3.0. The negative branch is below 0.

**Figure 2:**
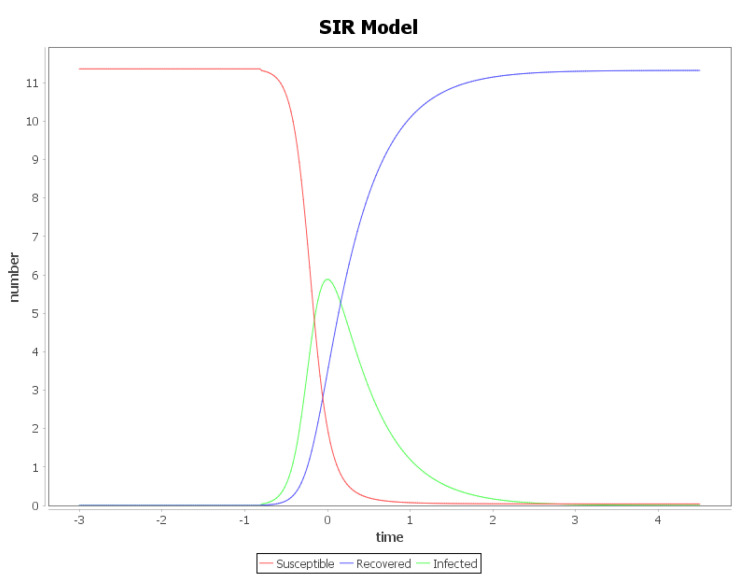
The SIR model variables as functions of time. The instance is parametrized by $i_{m}=5.81$, g=2.0.

Plots of the branches of the integral I(x) (Figure 1) were obtained by direct numerical integration using the QUADPACK [17] routines in the Computer Algebra System Maxima. Plots of the SIR model (Figure 2) were obtained using a Java routine [18] implementing the double exponential integration method [19,20]. Both methods turn out to be suitable for the numerical integration problem.

## 7. Datasets

The COVID datasets were downloaded from the European Centre for Disease Prevention and Control (ECDC) website: https://opendata.ecdc.europa.eu/covid19/casedistribution/csv. The downloadable data file was updated daily until 14 December 2020 and contains the latest available public data on COVID-19 aggregated per country. The data collection policy is available from https://www.ecdc.europa.eu/en/covid-19/data-collection.

## 8. Data Analysis Pipeline

The SIR model was formulated to model epidemic outbreaks with short incubation period [1]. The model has only two independent variables and two parameters, which allow for their estimation from raw data. This allows for efficient curve-fitting approach as demonstrated further.

The SIR model can be modified into SIRD to account for the deceased population. This modification will not result in qualitative difference in the observed dynamics as it does not change the structure of the equations for the *i* and *s*-variables. Hence, it allows for an analysis using the SIR model.

### 8.1. Data Processing

The data were imported in the SQLite https://www.sqlite.org database, filtered by country and transferred to MATLAB for parametric fitting using native routines. Quadratures were estimated by the default MATLAB integration algorithms Estimated parameter values were stored in the same database.

### 8.2. Parametric Fitting

The parametric fitting was conducted using the fminsearchbnd routine which implements least-squares constrained optimization algorithm and is an alternative to the proprietary non-linear least squares routine of MATLAB [21]. To reduce the impact of the fluctuation in the weekly reporting of data the parametric fitting procedure was applied first to three day moving average of the time series. The fitting equation is given by

$$I_{t}∼N\cdot i\left(t/10.0-T|g,i_{m}\right)$$

where $I_{t}$ is the observed incidence or case fatality, respectively. For numerical stability reasons the time variable was rescaled by a factor of 10. The $i_{m}$ and *T* were estimated from the observed data as the apparent peak and the time to the apparent peak, respectively. The initial estimate of *N* was fixed to 1. An initial estimate of 0.75 was used (i.e., corresponding to $R_{0}=1.33$) for the *g* parameter.

## 9. Case Studies

The approach in the present paper is exemplified with data from ECDC for several European countries in the period January 2020–December 2020. The data analysis is limited to 14 December, since then ECDC changed reporting policy to weekly averages instead of daily averages. The dataset is available from https://zenodo.org/record/4383118 [22]. The paper focuses on five European countries – Bulgaria, Belgium, the Netherlands, Germany and Italy for the following reasons. Italy was selected as the country where the first outbreak erupted in Europe. Germany was selected as another example of a large European country with very intense internal and cross-border traffic. Belgium and the Netherlands were selected as densely populated European countries with intense internal and cross-border traffic. Moreover, the outbreaks in both countries could be traced to the North Italian outbreak. Finally, Bulgaria was selected as an example of an European country with relatively few cases during the first half of 2020.

### 9.1. Analysis of Case Fatality Data

**Figure 3:**
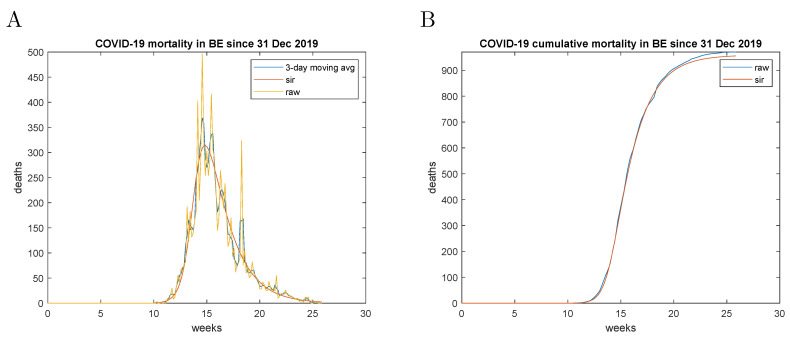
Case fatality model for Belgium. (**A**)—The parametric fit of the case fatality data; (**B**)—Cumulative deaths compared to the estimate from the r-variable. The origin corresponds to 1 January 2020.

**Figure 4:**
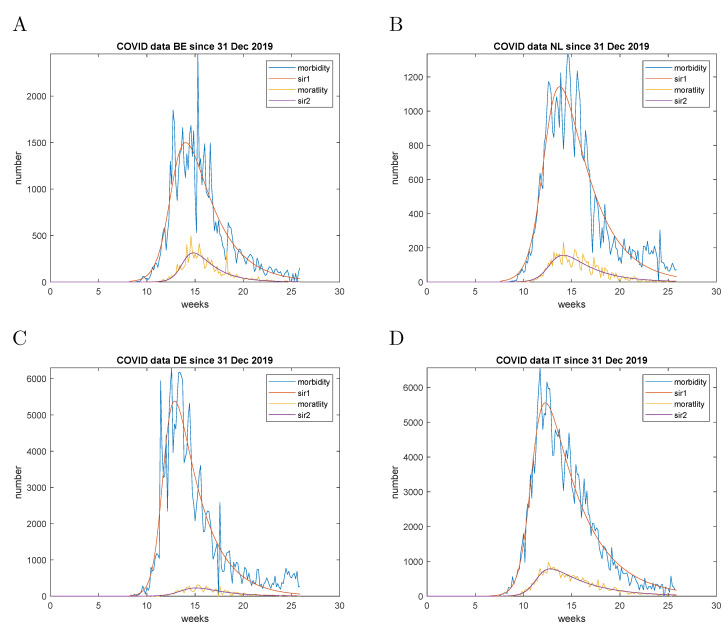
Incidence and case fatality modelling of the first wave. (**A**)—combined data for Belgium by 29 June 2020; (**B**)—combined data for The Netherlands by 29 June 2020; (**C**)—combined data for Germany by 29 June 2020; (**D**)—combined data for Italy by 29 June 2020; The raw data are smoothed with a 3-day moving average filter; ’mortality’ represents the reported case fatality, ’morbidity’ represents the reported incidence.

The observed case fatality represented a parameter, which could be established with more confidence in the beginning of the pandemic due to the lack of testing and the non-specificity of the clinical signs of COVID-19. Hence, it was the primary target of the parametric fitting. The data fitting procedures are illustrated with the case fatality data of Belgium and are presented in Figure 3. The intermediate parameters are initially estimated on the 3-day moving average data. The final fit was performed on the raw data, using the intermediate parameters as initial values. The cumulative mortality data were estimated from Equation (17). As can be appreciated from Figure 3 fluctuations in reporting did not have a detrimental effect on the estimation procedure. The results for Belgium, the Netherlands, Germany and Italy are presented in Table 1 and Figure 4.

### 9.2. Analysis of Incidence Data

The raw data demonstrated weekly fluctuations most probably caused by the reporting irregularity due to weekend breaks and fluctuations in the testing demand. It is especially pronounced for the morbidity data of Germany for the presented period. The incidence data were analysed in the same way using the fitted parameters for the case fatality as initial values. The fitting results demonstrate different lags of the peaks of incidence vs case fatality in the studied countries. For example, for Germany it was 2.2 weeks, while for the Netherlands it was 0.3 weeks.

### 9.3. Analysis of the Second Wave

**Figure 5:**
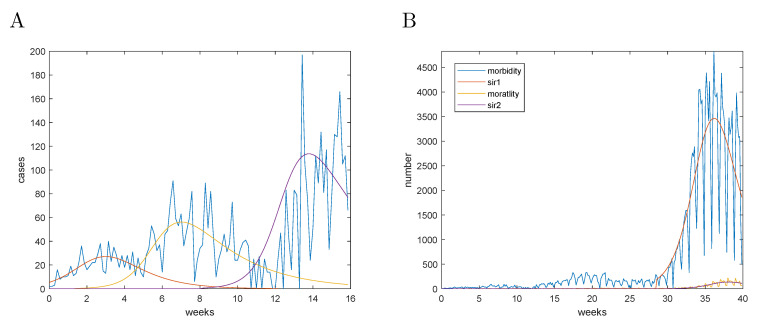
Modelling of consecutive outbreaks in Bulgaria. (**A**)—Raw data are compared to three fitted outbreaks recorded in the period 8 March 2020–29 June 2020. The origin corresponds to 8 March 2020 when the first COVID-19 case was reported. (**B**)—Raw data for the period 8 March 2020–14 Deccember 2020 were fitted against the SIR model; ‘sir1’—data fit of the reported cases; ‘sir2’—data fit of the reported deaths.

Changes of containment policies are meant to result in changes in the epidemic outbreak dynamics. This can be followed by the SIR model as demonstrated in the Bulgarian incidence dataset for the period March–July 2020, where resuming of public sports events in the end of June correlates with the 3rd increase of incidence (Figure 5). The process is difficult to automate because of the fluctuations in the data. Nevertheless, it was possible to accurately track the first three outbreaks.

**Figure 6:**
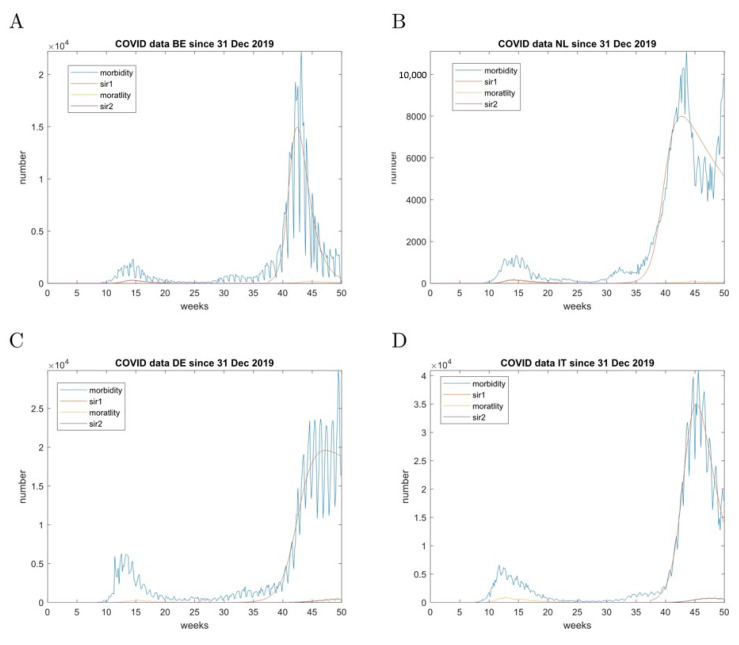
Incidence and case fatality modelling of the second wave. (**A**)—combined data for Belgium by 14 December 2020; (**B**)—combined data for The Netherlands by 14 December 2020; (**C**)—combined data for Germany by 14 December 2020; (**D**)—combined data for Italy by 14 December 2020; The raw data are smoothed with a 3-day moving average filter; mortality represents the case fatality, morbidity represents the incidence.

The same approach was applied in the analysis of the second pandemic wave generally appearing after week 20 in the reported dataset. The results are presented in Figure 6 and Table 2, Table 3 and Table 4. Bulgaria was added to Table 2, Table 3 and Table 4 since the data exhibited pronounced peaks in both case fatality and incidence. Regarding the case fatality, Belgium, the Netherlands, and Italy exhibited smaller peaks during the second wave. All countries (except Bulgaria, since there was no pronounced first wave, Figure 5) exhibited an increase of $R_{0}$ for the case fatality. The case fatality peaks exhibited synchrony for Belgium and the Netherlands, corresponding to their geographical proximity. This was also true for the incidence peaks.

Regarding the incidence, the peaks of the second wave were larger everywhere. Belgium and Italy exhibited a drop in $R_{0}$. The $R_{0}$ for Germany and the Netherlands appear to be anomalous as they are at large odds with the respective parameters for the case fatality.

## 10. Discussion

The presented results can be discussed along three main directions.

### 10.1. Analytical Aspects

For decades the SIR model evaded the efforts of the community to derive explicit solution. A formal analytical solution of the SIR model has been found only recently and formulated in the traditional setting of an initial value problem [13]. Yet, another perspective can be also of merit – the model can be treated as a manifold problem, which can be parametrized by any point on the flow. This is precisely the approach implemented by the peak-value parametrization in the present paper. The present paper establishes novel analytical results about the SIR model. The series solutions presented here are original. Other original results are the non-elementarity proofs for most of the presented integrals. Another interesting point is the proof of the non-Liouvillian character of the incidence *i*-function. An alternative proof could be also given, in principle, based on the work of Prelle and Singer [23].

### 10.2. Numerical Aspects

Presented results demonstrate the robustness of the fitting procedure with regard to the fluctuations in the raw data. On the other hand, more efforts are necessary in establishing a robust asymptotic of the incidence *i*-function. This is a clear direction for future research. Presented method is an alternative to commonly used phenomenological models (i.e., exponential, Richards, logistic, delayed logistic for $R_{0}$ estimation [24]) and can in principle be cast in a maximum likelihood fitting procedure. Such work, however, goes beyond the scope of the present paper.

### 10.3. Epidemiological Implications of the Results

Recorded data necessarily suffer from diverse biases. For example, the marked difference in the availability of tests in the early stages of the pandemics in different countries. Another one is the unsteady reporting resulting in fluctuations of the numbers. This severely limits the usefulness of the model formulation as an initial value problem, which is the standard numerical staring point. This can result in a drastic overestimation of the infection peak (see for example the predictions for UK in [25]).

A key finding of the present report is that simple models can be very useful in studying the epidemic outbreaks. This can be eventually extended to predicting the effects of different containment measures or the lack thereof [8]. Presented analysis corroborates the finding of other authors about the utility of the SIR model in analysing the COVID-19 pandemics [4,6,7,8]. Presented results indicate that there is a universality in the time evolution of COVID-19 and the same epidemic model, notably SIR, can be applied to countries having large differences in populations sizes and densities. More interestingly, the model seems to fit well also the case fatality data, which can be interpreted in the sense that the vulnerable population forms a distinct subpopulation from all susceptible individuals (e.g., elderly people). The data also lend support to a simple modification of the SIR model: notably – the SIRD model with an independent population of dead (D) persons. This corresponds to the recent findings of other authors [5,9].

## Figures and Tables

**Table 1:** Case fatality parameters, first wave. T is given in weeks and refers to the time passed since 1 January 2020.

Country	g	$R_{0}$	T [Weeks]	$i_{m}$
Belgium	0.7380	1.3549	14.8	313.97
Netherlands	0.5009	1.9962	14.1	156.72
Germany	0.6109	1.6370	15.1	226.51
Italy	0.3734	2.6760	12.8	785.39

**Table 2:** Case fatality parameters, second wave. T is given in weeks and refers to the time passed since 1 January 2020.

Country	g	$R_{0}$	T [Weeks]	$i_{m}$
Bulgaria	1.0	1.0	38.0	136.45
Belgium	0.4913	2.0356	44.8	201.88
Netherlands	0.3260	3.0679	45.3	71.00
Germany	0.5849	1.7096	49.8	408.33
Italy	0.3039	3.2906	47.6	731.09

**Table 3:** Incidence parameters, first wave. T is given in weeks and refers to the time passed since 1 January 2020; $R_{0}=1/g$; $i_{m}$ corresponds to the peak of the case fatality.

Country	g	$R_{0}$	T [Weeks]	$i_{m}$
Belgium	0.5500	1.8183	14.0	1499.59
Netherlands	0.5149	1.9420	13.8	1143.25
Germany	0.5483	1.8237	12.9	5383.26
Italy	0.4006	2.4961	12.3	5560.30

**Table 4:** Incidence parameters, second wave. T is given in weeks and refers to the time passed since 1 January 2020; $R_{0}=1/g$; $i_{m}$ corresponds to the peak of the case fatality.

Country	g	$R_{0}$	T [Weeks]	$i_{m}$
Bulgaria	1.1307	0.8844	46.2	3465.44
Belgium	1.0	1.0	42.4	14,970.01
Netherlands	0.1099	9.103	42.7	7995.74
Germany	0.0454	22.0382	47.3	19,595.36
Italy	0.5548	1.8024	45.2	34,949.74

## Appendix A. Supporting Results

**Proposition** **A1.**
*i(t) attains a global maximum $i=i_{m}=c-g\log g-g$.*

**Proof.** We use a parametrization for which $i\left(0\right)=i_{m}$. Then

$$\dot{i}\left(\tau \right)=-gi\left(W_{\pm }\left(-e^{\frac{i-i_{m}}{g}-1}\right)+1\right)$$

It follows that

$$\dot{i}\left(0\right)=0,\;i\left(0\right)=i_{m}$$

so $i_{m}$ is an extremum. In the most elementary way since W(z) should be real-valued then

$$-e^{\frac{i-i_{m}}{g}-1}\geq -1/e⟹\frac{i-i_{m}}{g}\leq 0$$

Hence, $i\leq i_{m}$. ☐

The proof of Theorem 1 establishes also the validity of two additional propositions:

**Proposition** **A2.**
*The integral*

$$I=\int \frac{dy}{y\left(g\log(y)-gy+c\right)}$$

*is not elementary.*

**Proposition** **A3.**
*The integral*

$$I=\int \frac{dy}{y+c-e^{y}}$$

*is not elementary.*

**Proof.** By change of variables $y=e^{x}$

$$I=\int \frac{dy}{y\left(\log(y)-y+c\right)}=-\int \frac{dx}{e^{x}-x-c}$$

☐

## Appendix B. Special Functions

### Appendix B.1. The Lambert W Function

The Lambert W function can be defined implicitly by the equation

$$W\left(z\right)e^{W(z)}=z,\;z\in C^{\;}$$

We observe that by Lemma A1 W(z) is transcendental. Furthermore, the Lambert function obeys the differential equation for x≠−1

$$W{\left(x\right)}^{\prime }=\frac{e^{-W(x)}}{1+W(x)}$$

W is a multivalued function. It has many applications in pure and applied mathematics. Properties of the W function are given in [11]. Useful identities

(A1) $$\begin{array}{cc} e^{-W(z)} & =\frac{W(z)}{z} \end{array}$$

(A2) $$\begin{array}{cc} e^{nW(z)} & ={\left(\frac{z}{W(z)}\right)}^{n} \end{array}$$

(A3) $$\begin{array}{cc} \log W(z) & =\log z-W(z) \end{array}$$

(A4) $$\begin{array}{cc} W\left(\frac{nz^{n}}{W{\left(z\right)}^{n-1}}\right) & =nW(z),\;n>0,z>0 \end{array}$$

The W function is non-elementary and in particular it is non-Liouvillian [26]. The latter fact is not widely known, as pointed out by the authors. Its indefinite integral is:

$$\int W\left(x\right)dx=xW\left(x\right)+\frac{x}{W(x)}-x$$

**Proposition** **A4.**

$$\int \frac{dy}{1+W\left(-\frac{e^{\frac{y-c}{g}}}{g}\right)}=g\log\left(-gW\left(-\frac{e^{\frac{y-c}{g}}}{g}\right)\right)+const$$

**Proof.** We differentiate

$$g{\left(\log W\left(-\frac{e^{\frac{y-c}{g}}}{g}\right)\right)}^{\prime }=-\frac{e^{\frac{y-c}{g}-W\left(-\frac{e^{\frac{y-c}{g}}}{g}\right)}}{gW\left(-\frac{e^{\frac{y-c}{g}}}{g}\right)\left(1+W\left(-\frac{e^{\frac{y-c}{g}}}{g}\right)\right)}=\frac{1}{1+W\left(-ge^{\frac{i-c}{g}}\right)}$$

☐

**Proposition** **A5.**

$$\int_{g\log g-g+c}^{i}\left.\frac{d\xi }{\xi \;\left(W_{\pm }\left(-\frac{e^{\frac{\xi -c}{g}}}{g}\right)+1\right)}\right.=\int_{g}^{-gW_{\pm }\left(-\frac{e^{\frac{i-c}{g}}}{g}\right)}\left.\frac{dy}{y\;\left(g\log y-y+c\right)}\right.$$

**Proof.** We use the change of variables ξ−c=glogy−y and then simplification by the defining identity of the Lambert W function.

$$\begin{array}{c} \int_{g\log g-g+c}^{i}\left.\frac{d\xi }{\xi \;\left(W\left(-\frac{e^{\frac{\xi -c}{g}}}{g}\right)+1\right)}\right.=\int_{A}^{B}\frac{\frac{g}{y}-1}{\left(g\log y-y+c\right)\;\left(W\left(-\frac{e^{\frac{g\log y-y+c}{g}-\frac{c}{g}}}{g}\right)+1\right)}dy=\\ \int_{A}^{B}\frac{g-y}{-\frac{y\;\left(gy\log y-y^{2}+cy\right)}{g}+gy\log y-y^{2}+cy}dy=g\int_{A}^{B}\frac{dy}{y\;\left(g\log y-y+c\right)} \end{array}$$

where

glogA−A+c=glogg−g+c, glogB−B+c=i

Therefore, A=g and $B=-gW\left(-\frac{e^{\frac{i-c}{g}}}{g}\right)$. ☐

### Appendix B.2. The Wright *Ω* Function

The Wright Ω function is related to the Lambert W function [11].

$$\Omega \left(z\right)=W_{K(z)}\left(e^{z}\right),\;z\in C$$

where $K\left(z\right)=\left\lceil \left(Im(z)-\pi \right)/2\pi \right\rceil$ is the unwinding number of *z*. Moreover,

Ω(z)+logΩ(z)=z, z≠t±iπ,t≤−1,

for the principal branch of the logarithm. It is a transcendental function. It obeys the differential equation

$$\Omega {\left(x\right)}^{\prime }=\frac{\Omega (x)}{1+\Omega (x)}$$

The Ω function is non-Liovillian [26]. Its indefinite integral is:

$$\int \Omega \left(x\right)dx=\frac{{\Omega (x)}^{2}}{2}+\Omega \left(x\right)+const$$

## Appendix C. Differential Fields

**Definition** **A1.**
*Denote by $C^{\;}\left(x,c_{i},\theta_{i}\right)$ the complex-valued ring, generated by the finite set of rational functions ${\left\{\theta_{i}\right\}}_{i}^{n}$ and constants ${\left\{c_{i}\right\}}_{i}^{n}$.*

**Definition** **A2.**
*An element θ is called algebraic if P(x,θ)=0 for some polynomial*

$$P\left(x,t\right)=t^{m}+a_{m-1}t^{m-1}+\ldots +a_{0},$$

*where $a_{i}$ can be also rational functions of x, or else it is called transcendental.*

**Lemma** **A1** (Composition lemma)**.** *Denote by a and t the algebraic or transcendental elementary functions, respectively. The following compositions hold*

a∘a=a, t∘a=t, a∘t=t

**Proof.** The case a∘a when a(x) is a polynomial is trivial. Suppose that a and b are both algebraic:

P(x,a)=0, Q(x,b)=0

Without loss of generality suppose that $a_{i}$ are polynomial. Formally, $b={\bar{f}}_{k}\left(x\right)$ for any branch *k* with $\bar{f}$ algebraic since it is a root of a polynomial, where the bar denotes the inverse function in order to avoid confusion with exponentiation. Therefore,

$$a\circ b=b^{m}+a_{m-1}b^{m-1}+\ldots +a_{0}={\bar{f}}_{k}^{m}\left(x\right)+a_{m-1}{\bar{f}}_{k}^{m-1}\left(x\right)+\ldots +a_{0}$$

is algebraic since it is computed by a finite sequence of algebraic operations. Suppose that t=exp(x). Then exp(a) is not algebraic, hence it is transcendental.

$$P\left(x,e^{x}\right)=e^{xm}+a_{m-1}e^{xm-x}+\ldots +a_{0}$$

is exponential. Suppose that t=log(x). Then log(a) is not algebraic, hence it is transcendental.

$$P\left(x,\log\left(x\right)\right)=\log^{m}x+a_{m-1}\log^{m-1}x+\ldots +a_{0}$$

is not algebraic, hence it is transcendental. ☐

In what follows is assumed that the differential field is of characteristic zero and has an algebraically closed field of constants. An element y of a differential field is said to be an *exponential* of an element *A* if $y^{\prime }=Ay$, an exponential of an integral of an element *A* if $y^{\prime }=Ay$; logarithm of an element *A* if $y^{\prime }=A^{\prime }/A$, and an integral of an element *A* if $y^{\prime }=A$.

The next definition is due to [26].

**Definition** **A3.**
*Let $\left(k{,}^{\prime }\equiv d/dx\right)$ be a differential field of characteristic 0. A differential extension $\left(K{,}^{\prime }\equiv d/dx\right)$ of k is called Liouvillian over k if there are $\theta_{1},\;\ldots ,\;\theta_{n}\in K$, such that $K=C(x,\theta_{1},\;\ldots ,\;\theta_{n})$ and for all i, at least one of the following*

1. *$\theta_{i}$ is algebraic over $k(\theta_{1},\;\ldots ,\;\theta_{n-1})$*
2. $\theta_{i}^{\prime }\in k\left(\theta_{1},\;\ldots ,\;\theta_{n-1}\right)$
3. $\theta_{i}^{\prime }/\theta_{i}\in k\left(\theta_{1},\;\ldots ,\;\theta_{n-1}\right)$

*holds. The constant subfield C(K) of K is defined to be the set of c in K, such that $c^{\prime }=0$.*

The next theorem is due to [16].

**Theorem** **A1.**
*If K is an elementary field, then it is closed under differentiation.*

An elementary integrability theorem due to Conrad [16].

**Theorem** **A2** (Rational Liouville criterion)**.** *For $f,g\in C^{\;}\left(x\right)$ with f and g non-constant the function $f\left(x\right)e{}^{g}\left(x\right)$ can be integrated in elementary terms if and only if there exists a rational function $h\in C^{\;}\left(x\right)$ such that $h^{\prime }+g^{\prime }h=f$.*

The last result can be extended to algebraic functions as follows.

**Corollary** **A1** (Algebraic Liouville criterion)**.** *For f(x),g(x) algebraic and non-constant, the function $f\left(x\right)e^{g(x)}$ can be integrated in elementary terms if and only if there exists an algebraic function h(x), for which $h^{\prime }+g^{\prime }h=f$.*

**Proof.** Suppose that *f* and *g* are arbitrary elementary algebraic functions. Denote the primitive of *f* as f÷F. The integral can be integrated by parts

$$I=\int f\left(x\right)e{}^{g(x)}dx=\int e^{g}\left(x\right)dF=F\left(x\right)e^{g(x)}-\int F\left(x\right){\left(e^{g(x)}\right)}^{\prime }dx$$

Therefore,

$$\int \left(f\left(x\right)+F\left(x\right)g^{\prime }\left(x\right)\right)e^{g(x)}dx=F\left(x\right)e^{g(x)}$$

We observe that $g^{\prime }\left(x\right)$ is elementary by Theorem A1. The L.H.S has the form $fe^{g}$ and since $f\left(x\right)+F\left(x\right)g^{\prime }\left(x\right)$ is elementary we can identify

$$h\equiv F,\;f_{1}\equiv f+Fg^{\prime }=h^{\prime }+hg^{\prime }$$

so that $\left(h^{\prime }+hg^{\prime }\right)e^{g}={\left(he^{g}\right)}^{\prime }$ and the claim follows. ☐
